# X Chromosome Control of Meiotic Chromosome Synapsis in Mouse Inter-Subspecific Hybrids

**DOI:** 10.1371/journal.pgen.1004088

**Published:** 2014-02-06

**Authors:** Tanmoy Bhattacharyya, Radka Reifova, Sona Gregorova, Petr Simecek, Vaclav Gergelits, Martin Mistrik, Iva Martincova, Jaroslav Pialek, Jiri Forejt

**Affiliations:** 1 Mouse Molecular Genetics Group, Division BIOCEV, Institute of Molecular Genetics, Academy of Sciences of the Czech Republic, Prague, Czech Republic; 2 Institute of Molecular and Translational Medicine, Faculty of Medicine and Dentistry, Palacký University Olomouc, Olomouc, Czech Republic; 3 Research Facility Studenec, Institute of Vertebrate Biology, Academy of Sciences of the Czech Republic, Brno, Czech Republic; University of Wisconsin–Madison, United States of America

## Abstract

Hybrid sterility (HS) belongs to reproductive isolation barriers that safeguard the integrity of species *in statu nascendi*. Although hybrid sterility occurs almost universally among animal and plant species, most of our current knowledge comes from the classical genetic studies on *Drosophila* interspecific crosses or introgressions. With the house mouse subspecies *Mus m. musculus* and *Mus m. domesticus* as a model, new research tools have become available for studies of the molecular mechanisms and genetic networks underlying HS. Here we used QTL analysis and intersubspecific chromosome substitution strains to identify a 4.7 Mb critical region on Chromosome X (Chr X) harboring the *Hstx2* HS locus, which causes asymmetrical spermatogenic arrest in reciprocal intersubspecific F1 hybrids. Subsequently, we mapped autosomal loci on Chrs 3, 9 and 13 that can abolish this asymmetry. Combination of immunofluorescent visualization of the proteins of synaptonemal complexes with whole-chromosome DNA FISH on pachytene spreads revealed that heterosubspecific, unlike consubspecific, homologous chromosomes are predisposed to asynapsis in F1 hybrid male and female meiosis. The asynapsis is under the *trans*- control of *Hstx2* and *Hst1/Prdm9* hybrid sterility genes in pachynemas of male but not female hybrids. The finding concurred with the fertility of intersubpecific F1 hybrid females homozygous for the *Hstx2^Mmm^* allele and resolved the apparent conflict with the dominance theory of Haldane's rule. We propose that meiotic asynapsis in intersubspecific hybrids is a consequence of *cis*-acting mismatch between homologous chromosomes modulated by the *trans*-acting *Hstx2* and *Prdm9* hybrid male sterility genes.

## Author Summary

Genomes of newly emerging species restrict their gene exchange with related taxa in order to secure integrity. Hybrid sterility is one of the reproductive isolation mechanisms restricting gene flow between closely related, sexually reproducing organisms. We showed that hybrid sterility between two closely related mouse subspecies is executed by a failure of meiotic synapsis of orthologous chromosomes in F1 hybrid males. The asynapsis of orthologous chromosomes occurred in meiosis of male and female hybrids, though only males were sterile due to *trans*-acting male-specific hybrid sterility genes. We located one of the two major hybrid sterility genes to a 4.7 Mb interval on Chromosome X, showed that it controls male sterility by modulating the extent of meiotic asynapsis and using the inter-subspecific chromosome substitution strains we refuted the simple interpretation of dominance theory of Haldane's rule. A new working hypothesis posits male sterility of mouse inter-subsubspecific F1 hybrids as a consequence of meiotic chromosome asynapsis caused by the *cis*-acting mismatch between orthologous chromosomes modulated by the *trans*-acting hybrid male sterility genes.

## Introduction

Hybrid sterility (HS) is a postzygotic reproductive isolation barrier restricting gene flow between the related taxa during speciation. It is defined as a condition where two parental forms fertile *inter se* produce a hybrid that is sterile [1]. One of the most interesting findings coming from previous studies is a disproportionately large effect of Chr X on reproductive isolation, particularly on hybrid sterility and inviability. The large X-effect was described in diverse organisms, and evolutionary biologists designated it as one of the speciation rules [2]–[5]. Another speciation principle, called Haldane's rule [6], points to the empirical findings that hybrid inviability and sterility predominantly afflicts the heterogametic (XY or ZW) sex. The dominance theory originally proposed by Muller [7] explained the sex-dependent effect on hybrid fitness by the manifestation of recessive X-linked alleles in hemizygous XY males but not in XX females [8]–[10].

We have chosen *M. m. musculus* and *M. m. domesticus* subspecies (hereafter, *Mmm* and *Mmd*) as model organisms to study mammalian HS (for review see [11]–[13]). Both subspecies diverged from a common ancestor approximately 0.3 to 0.5 million years ago [14] and formed a hybrid zone across Europe after their secondary contact [15]. The repeated introgressions of *Mmm* genes into *Mmd* genome and *vice versa* across their hybrid zone indicate incomplete reproductive isolation between both young subspecies [16], [17]. Such early-stage model is superior in that it reduces the risk of analyzing HS genes that evolved as a consequence and not as the cause of speciation after full reproductive isolation of the related taxa [5], [18]. Numerous genetic and genomic tools are available for the mouse model, including the full genomic sequence of inbred strains representing both subspecies and additional 17 laboratory inbred strains [19] and a panel of 28 mouse intersubspecific chromosome substitution (consomic) strains carrying individual *Mmm* chromosomes or their parts on *Mmd* background [20]. A variety of commercially available antibodies detecting meiosis-specific proteins and histone modifications permit immunodetection of subnuclear structures important for meiotic chromosome synapsis and segregation [21], [22].

We identified the first hybrid sterility gene in mice, hybrid sterility 1 – *Hst1* – as a polymorphic variant on Chr 17 between two laboratory strains, C57BL10/Sn and C3H/Di, both predominantly of *Mmd* origin (at that time still linkage group IX). When mated with *Mmm* wild mice trapped in Central Bohemia near Prague, these crosses produced sterile or fertile male hybrids, depending on their *Hst1* alleles [23]. Recently, *Hst1* was identified by the forward genetics approach as PR domain containing 9 (*Prdm9*) [24] and later was shown to control meiotic recombination hotspots [25], [26].

In a study of genetic architecture of F1 hybrid male sterility, the results of (*Mmm*×*Mmd*)×*Mmd* backcross predicted a minimum of four independently segregating HS loci. However, QTL analysis of the data revealed only two strong HS loci, *Hst1/Prdm9* and a locus on Chr X [27]. This paradox could be explained either by the action of multiple minor HS loci undetected by relatively low-power QTL analysis or by different behavior of two major HS loci on the hybrid background. The latter alternative was supported in an experiment showing that these two HS loci are not sufficient to recapitulate the F1 HS phenotype on B6 (*Mmd*) genetic background [22]. Moreover, for the first time a compelling evidence was provided for the mechanism of HS, showing that aberrant meiotic pairing of heterosubspecific homologous chromosomes and meiotic arrest are the consequence of intersubspecific hybrid genetic background [22].

Here we report on the role of Chr X in male and female meiosis in mouse intersubspecific hybrids. We localized the *Hstx2* locus controlling the asymmetry of HS in reciprocal intersubspecific F1 hybrid males to a 4.7 Mb interval on Chr X and mapped three autosomal loci that can abolish this asymmetry. We observed the predisposition of heterosubspecific homologs to asynapsis in male and female meiosis as the initial step of intrameiotic breakdown of the sterile hybrids. The effects of *Hstx2* or *Hst1/Prdm9* on the degree of asynapsis in pachynemas of male but not female intersubspecific hybrids concurred with the fertility of *Mmm×Mmd* F1 hybrid females homozygous for the *Hstx2^Mmm^* allele and resolved the apparent conflict with the dominance theory of Haldane's rule. Based on detailed meiotic analysis of the mouse model of intersubspecific F1 hybrids we propose that HS genes operate on “sensitized” genetic background resulting from the difficulties of proper meiotic synapsis of heterosubspecific autosomal homologs and consequent epigenetic dysregulation of X-Y chromosomes [22]. Accordingly, classical HS genes *Prdm9* [24], [28] and *Hstx2* [27] realize their HS-specific phenotypes by interacting, directly or indirectly, with the process of meiotic pairing and synapsis of heterospecific homologs.

## Results

### Fine mapping of *Hstx2* and its role in HS asymmetry of reciprocal F1 hybrids

**Figure 1:**
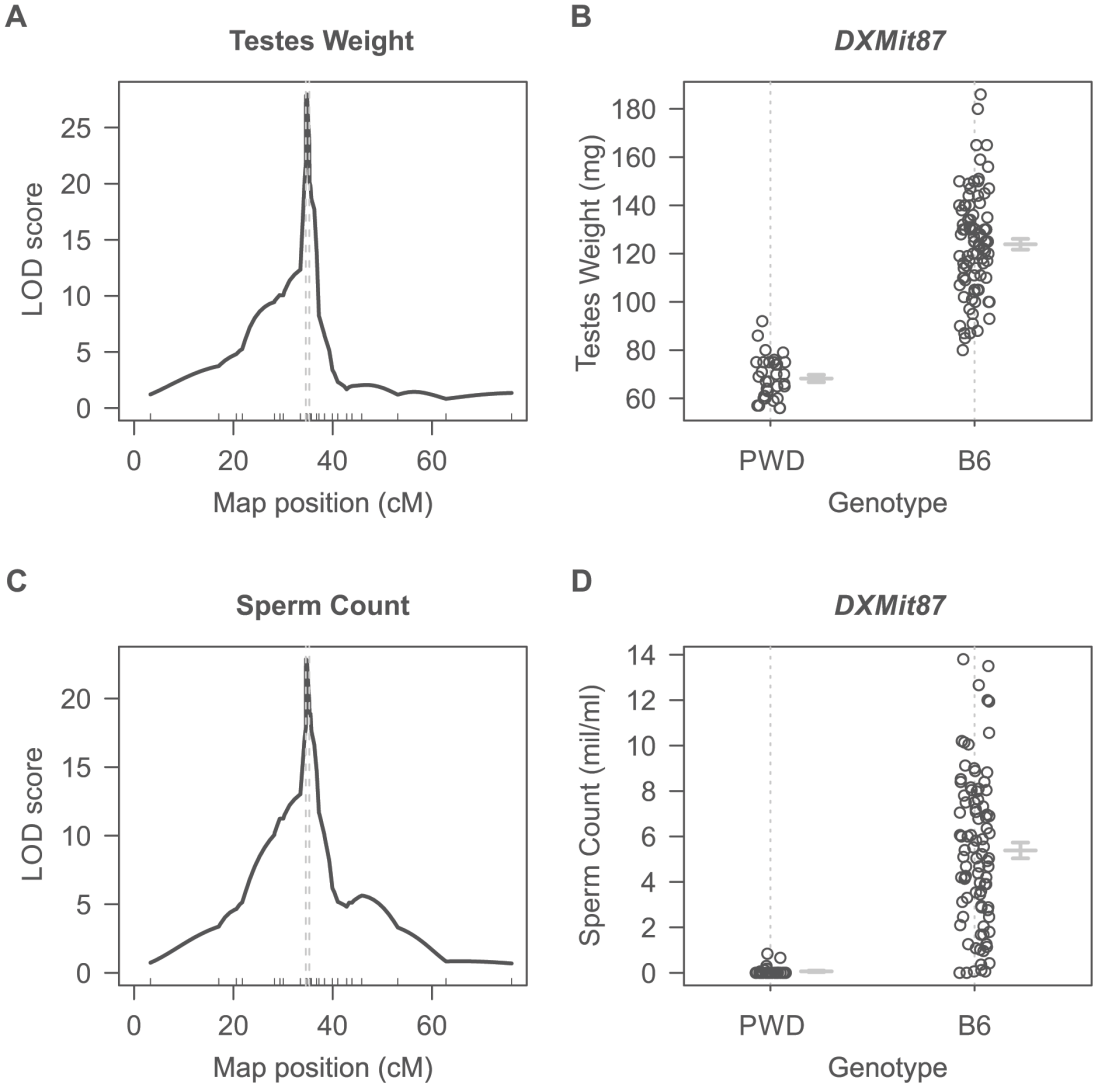
Single QTL mapping of *Hstx2* on Chr X. (A) QTL analysis of testes weight in the (B6.PWD-Chr X×B6)F1×PWD cross showed a 1.5-LOD support interval between 34.6 cM to 35.3 cM on Chr X. (B) Distribution of testes weight of males carrying PWD or B6 allele of *DXMit87* marker with LOD score 30. (C) QTL analysis of sperm count in *ductus epididymis* shows the same 1.5 LOD support interval as for testes weight. (D) Distribution of sperm count of males carrying PWD or B6 allele of *DXMit87* marker (Chr X: 66.65 Mb, GRCm38) with LOD score above 20.

Dobzhansky-Muller incompatibilities (DMIs) between the nascent species often result in asymmetry of HS or inviability of reciprocal F1 hybrids [27], [29]–[31] although relatively little is known about their genetic control or mechanistic basis. Previously we have shown that asymmetry in male sterility of reciprocal hybrids between *Mmm* mouse subspecies represented by the PWD/Ph (hereafter PWD) inbred strain [32] and *Mmd* represented by the C57BL/6J inbred strain (hereafter B6) is controlled by the central region of Chr X (64.9 Mb–98.1 Mb, GRCm38). To localize the locus responsible for HS asymmetry we crossed consomic F1 females (B6.PWD-Chr X×B6) with PWD males. All 124 male offspring carried B6/PWD heterosubspecific autosomal pairs while Chr X loci were either PWD or B6, depending on the recombination breakpoints. Testes weight (TW, range 56–186 mg) and sperm count (SC, 0–13.5 million) were used as surrogate for QTL analysis of male fertility phenotypes. Their segregation localized a hybrid sterility locus to the 34.6–35.7 cM (1.5-LOD support interval) interval with the maximum LOD score 30 and 23 at 34.8 cM for TW and SC. As shown in Fig. 1A–D all males that received the PWD allele at the *DXMit87* locus had small testes bellow 100 mg and little (below 10^6^) sperm in *ductus epididymis*. This hybrid sterility locus causes complete sterility on F1 hybrid intersubspecific background, and we designate it hybrid sterility chromosome X 2, *Hstx2*. Earlier, we reported localization of the locus at lower resolution in the (PWD×B6)×B6 backcross (Dzur-Gejdosova 2012).

To further refine the position of *Hstx2*, a new partial consomic strain B6.PWD-Chr X.1s was created with extended proximal interval of the Chr X^PWD^ sequence compared to B6.PWD-Chr X.1 (for strain description see ref [20]).

**Figure 2:**
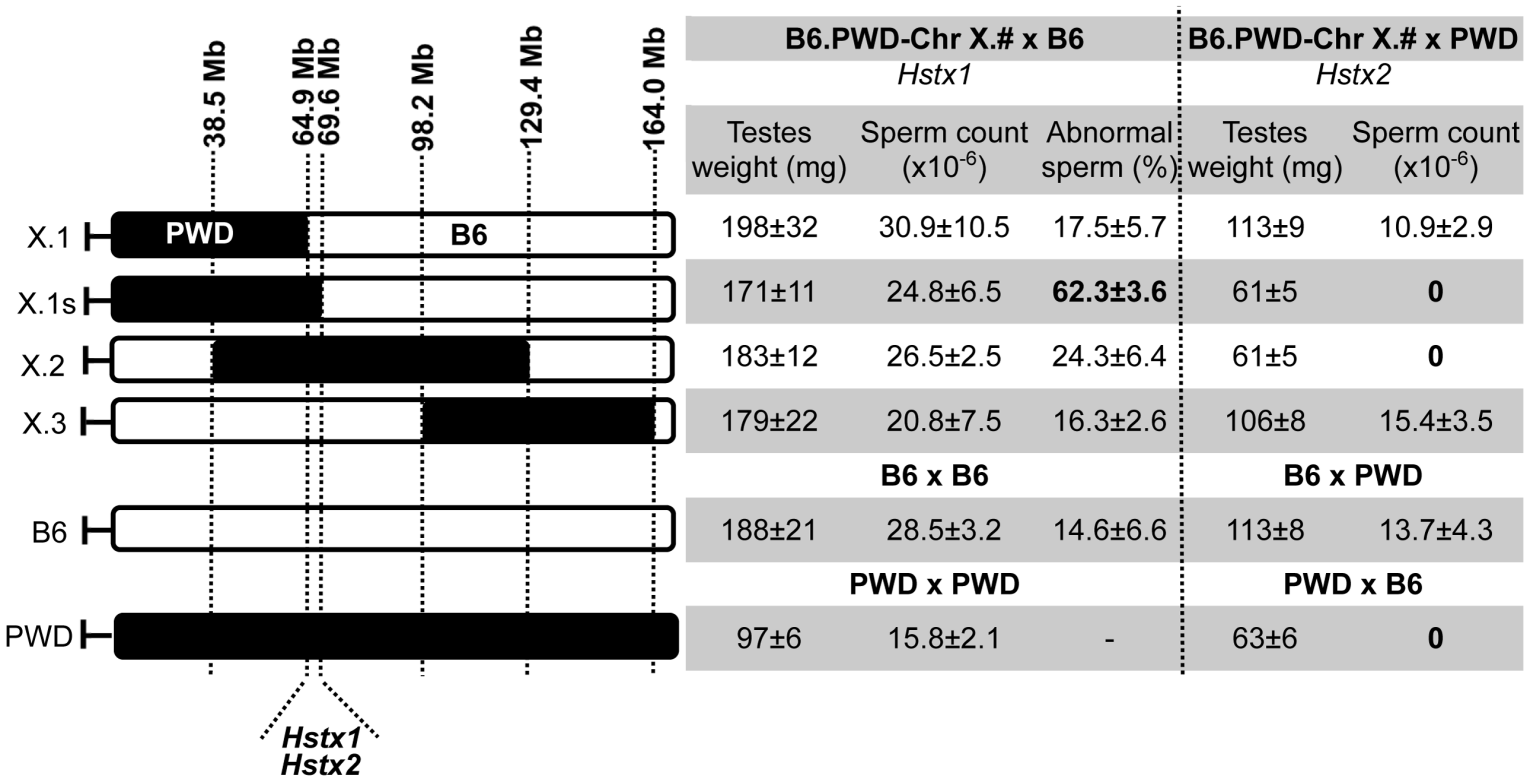
Fine mapping of *Hstx1* and *Hstx2* HS loci on Chr X using partial consomic strains. The partial consomic B6.PWD-Chr X.# females were crossed with B6 or PWD males for mapping *Hstx1* and *Hstx2*, respectively. Testes weight and sperm count were used as fertility phenotypes. The borders of introgressed PWD sequence (black) were determined by MegaMUGA genotyping for B6.PWD-Chr X.1 (abbreviated here X.1), B6.PWD-Chr X.1s (X.1s) and B6.PWD-Chr X.2 (X.2). For B6.PWD-Chr X.3 (X.3) mapping see [20]. The map positions correspond to genome assembly GRCm38, megabase scale, for details see Table S1.

The borders of the PWD sequence of the introgressed Chr X^PWD^ were compared to those of the existing proximal, central and distal partial consomic strains B6.PWD-Chr X.1, B6.PWD-Chr X.2 and B6.PWD-Chr X.3 [20] using high-resolution Mouse Universal Genotyping Array (MegaMUGA) (Table S1). To localize the region carrying *Hstx2* on Chr X^PWD^, females of all four Chr X partial consomic strains were crossed with PWD males and the fertility of male offspring was examined (Fig. 2). The (B6.PWD-Chr X.1×PWD)F1 and (B6.PWD-Chr X.3×PWD)F1 hybrid males were semifertile with testes weight comparable to (B6×PWD)F1 males, while (B6.PWD-Chr X.1s×PWD)F1 and (B6.PWD-Chr X.2×PWD)F1 were fully sterile with small testes (p<0.0001, t-test) and no sperm in *ductus epididymis* (Fig. 2). Thus (B6.PWD-Chr.X.1s×PWD)F1 hybrids carried the *Hstx2^PWD^* allele and fully reconstructed the HS phenotype of (PWD×B6) males, showing meiotic arrest at epithelial stage IV and to lesser degree at late pachytene/diplotene stage (Table S2). The position of *Hstx2* was localized to the 4.7 Mb interval delineated by UNC30904273 for the distal end of B6.PWD-Chr X.1 PWD sequence and UNC30934795 for the distal end of B6.PWD-Chr X.1s (X: 64,880,641–69,581,094, GRCm38).

### Introgressed *Hstx1^PWD^* causes teratozoospermia in *Mmd* genome and maps to the same genomic region as *Hstx2*

The *Hstx1* locus in the proximal part of Chr X^PWD^ causes male sterility when introgressed onto the B6 background [33]. Contrary to the F1 hybrid meiotic arrest at late pachytene/diplotene stage controlled by *Hstx2* [22], *Hstx1^PWD^* in the B6 genome causes postmeiotic breakdown and abnormal morphology of a fraction of non-functional sperm. Here we genotyped the male progeny of females heterozygous for PWD and B6 form of Chr X from the backcross generations 4–9 to B6 background and selected 71 Chr X single-recombinants with PWD centromeric end for fertility testing. The *Hstx1* locus mapped within the interval spanned by *DXMit76* and *DXMit143* (Fig. S1).

To further localize *Hstx1*, we phenotyped all four B6.PWD-Chr X partial consomics and found a high percentage of abnormal sperm cells in B6.PWD-Chr X.1s compared to other three partial consomics and B6 males (p<0.05 t-test, Fig. 2). The analysis of partial consomic strains independently confirmed the *Hstx1* localization to the same 4.7 Mb interval of Chr X that carries *Hstx2*. However, because B6.PWD-Chr X.2 males did not show high frequency of abnormal sperm in spite of their *Hstx1^PWD^* allele, we assumed that *Hstx1* needs to interact with another genetic factor from the proximal region of Chr X^PWD^ to manifest the abnormal sperm phenotype (Fig. 2, see also [33]).

### *Hstx1* and *Hstx2* candidate genes

**Figure 3:**
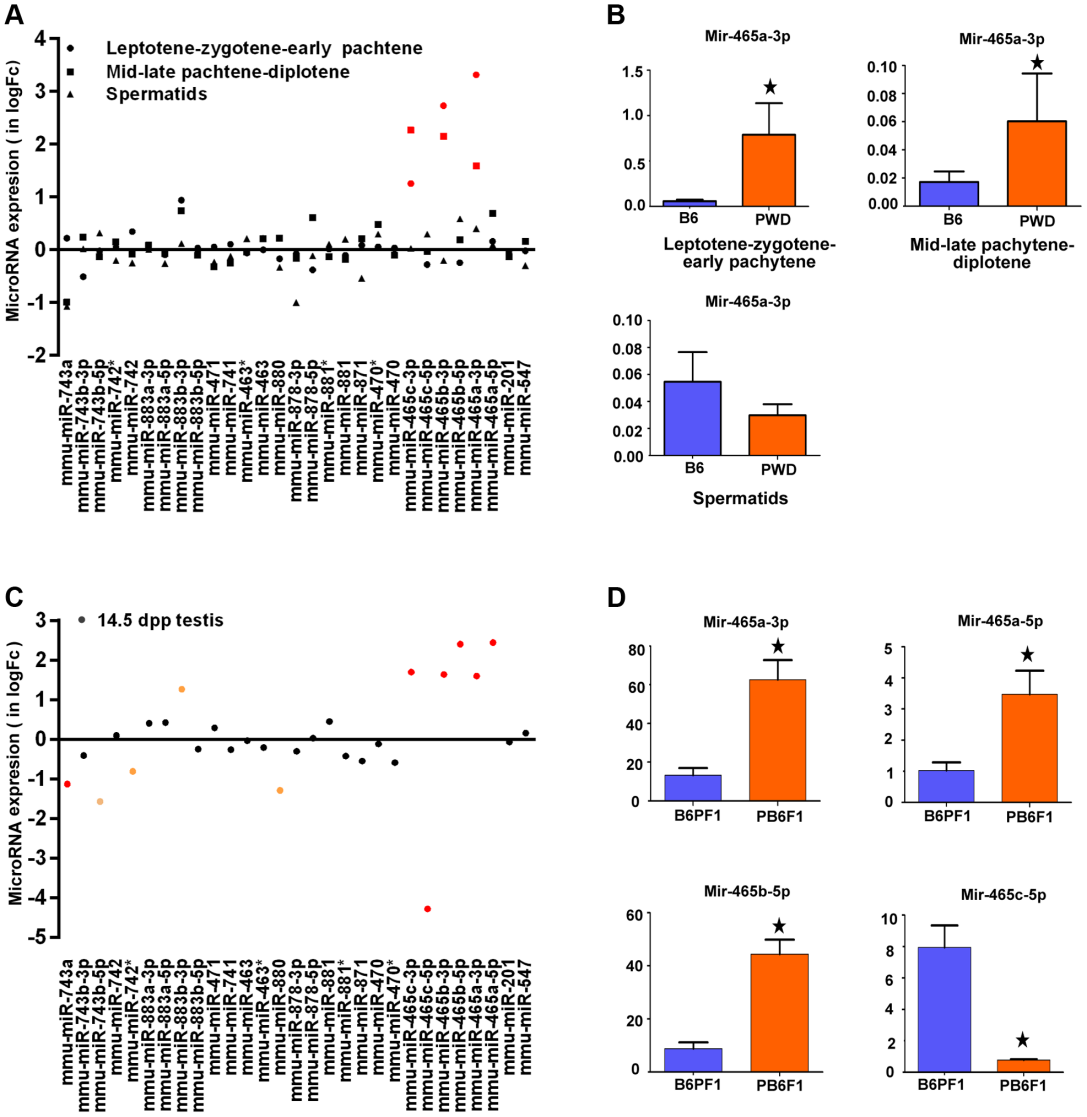
Expression profiling of a cluster of MiRNA genes within the *Hstx1/Hstx2* critical region. (A) Log fold-change of expression ratio of PWD versus B6 MiRNA genes in flow-sorted testicular cells. Significant overexpression of Mir465 cluster in PWD germ cells is shown in red. (B) Validation of Mir465 overexpression in PWD primary spermatocytes by qRT PCR. Data normalized to U6 non-coding RNA (C) Log fold-change of expression ratio of MiRNA genes in (PWD×B6)F1 versus (B6×PWD)F1 (abbreviated PB6F1 and B6PF1) 14.5 d old testes. Significant upregulation in red (P<0.05). (D) qRT PCR validation of differences in Mir465 expression. Data normalized to Mir152.

The 4.7 Mb Chr X candidate region of *Hstx1* and *Hstx2* loci carries 11 known protein-coding genes and 20 miRNA genes (Fig. S2). Of these, seven protein-coding genes, namely cancer/testis antigen 2 (C*tag2*), RIKEN cDNA 4930447F04 gene (*4930447F04Rik*), SLIT and NTRK-like family, member 2 (*Slitrk2*), RIKEN cDNA 4933436I01 gene (*4933436I01Rik*), fragile X mental retardation syndrome 1 homolog (*Fmr1*), fragile X mental retardation 1 neighbor (*Fmr1nb*) and AF4/FMR2 family member 2 (*Aff2*) show high expression in adult testis. Of them, *Aff2* is expressed pre-meiotically in spermatogonia, *Fmr1* and *Fmr1nb* show expression in early prophase I, and *Ctag2*, *4930447F04Rik* and *Slitrk2* are expressed in meiotic and postmeiotic cells. Finally, *4933436I01Rik* is expressed in haploid cells. Sorted populations of testicular cells from PWD and B6 strains and immature 14.5 dpp testes of their reciprocal hybrids did not show significant differences in relative mRNA expression levels of six meiotic and/or postmeiotic genes (Fig. S3). All 20 miRNAs in the candidate region are expressed in male germ cells but do not undergo meiotic sex chromosome inactivation (MSCI) [34]). Only the Mir465^PWD^ cluster displayed a significant increase of expression in the first meiotic prophase of PWD and B6 sorted testicular cells (Figure 3A, B). The miRNA expression profiling of sterile (PWD×B6)F1 and fertile (B6×PWD)F1 14.5dpp testes showed 1.5- to 2-fold upregulation of *Mir883b-3p*, *Mir465a/b/c-3p* and *Mir465a/b -5p* in sterile males, while *Mir743a*, *Mir743-5p*, *Mir880* and *Mir465c-5p* showed 1.2- to 4-fold down-regulation (Figure 3C, D).

Re-sequencing candidate genes from PWD genomic DNA and BAC clones [35] and inspection of the PWD exome sequence revealed seven non-synonymous substitutions of the *4933436I01Rik* PWD allele compared to B6 (see also [36]). Of the remaining genes, *Aff2* carries five, *Fmr1nb* and *Slitrk2* carry two, *4930447F04Rik* and C*tag2* carry one, and *Fmr1* does not carry any non-synonymous substitution. Inspection of Sanger Institute Mouse Genome Project (http://www.sanger.ac.uk/resources/mouse/genomes/) confirmed the same SNPs for PWK, a closely related *Mmm* inbred strain. Search of miRNA sequences revealed one SNP in the seed sequence of *Mir743a*, changing AAAGACA in B6 to AAAGACG in PWD (Table 1).

**Table 1:** Missense SNPs of candidate genes in the *Hstx1/Hstx2* critical region on Chr X.

Gene Symbol	SNP position^a^	B6	PWD	PWK	dN∶dS^b^
*Ctag2*	65 047 953	T	G	G	0.57
*4930447F04Rik*	66 303 564	A	C	C	0.54
*Slitrk2*	66 655 874	A	G	G	0.11
	66 656 111	A	G	G	
*Mir743*	66 776 774	T	C	C	-
*4933436I01Rik*	67 920 137	C	T	T	0.82
	67 920 143	T	G	G	
	67 920 312	G	T	T	
	67 920 431	T	A	A	
	67 920 805	T	A	A	
	67 920 818	C	T	T	
	67 920 822	T	G	G	
*Fmr1*	-	-	-	-	0.06
*Fmr1nb*	68 762 025	C	G	G	0.65
	68 769 064	T	A	A	
*Aff2*	69 544 913	A	G	G	0.20
	69 830 745	C	T	T	
	69 830 760	C	G	G	
	69 830 782	A	T	T	
	69 834 780	G	A	A	

a SNPs for protein coding genes were extracted from the PWD exome sequence compared to B6 reference genome (GRCm38) and confirmed by classical re-sequencing. PWK SNPs were obtained from Sanger Institute Mouse Genome Project.

b Rate of protein evolution is based on one to one comparison with rat orthologs. (GRCm37).

Reproductive isolation genes in *Drosophila* and in mouse have been shown to evolve rapidly and to undergo positive selection [37]–[39]. Three of the candidates for the *Hstx1*/*2* locus, *Ctag2*, *4933436I01Rik* and *Fmr1nb*, displayed an elevated rate of protein evolution (Table 1). In particular, *4933436I01Rik* is among the most rapidly evolving genes on Chr X [40], [41], showing weak but significant expression in primary spermatocytes and strong post-meiotic expression [42], https://www.genevestigator.com/gv/). *Fmr1nb*, another possible candidate, shows high expression in pre-pachytene spermatocytes [43].

### Intrasubpecific autosomal polymorphisms suppress asymmetry of HS

**Figure 4:**
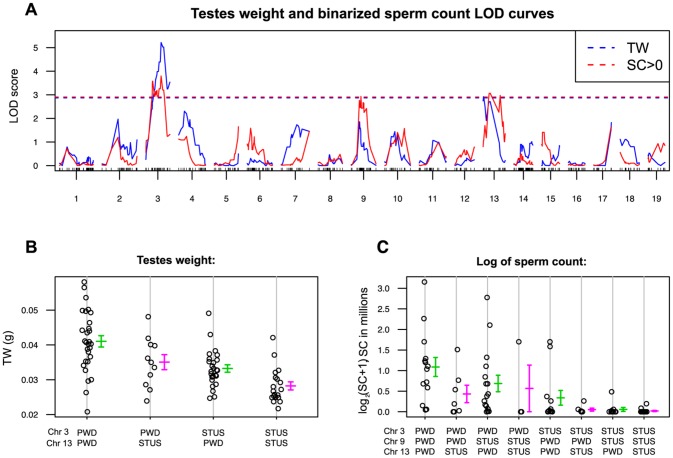
Single QTL scan for autosomal loci supporting *Hstx2* independent intrameiotic arrest of (*Mmd*×*Mmm*)F1 intersubspecific hybrids. (A) Testes weight QTLs (red) reached significance on Chrs 3 (marker UNC030163295, Chr3: 104,320,699) and 13 (JAX00357337 Chr 13:47,975,634) and QTLs for sperm count on Chrs 3, 9 (JAX00171568, Chr 9:56,491,601) and 13. Sperm count was evaluated as a binary trait (SC = 0, SC>0). (B) and (C) Additive effect of QTLs on testes weight and sperm count. For map positions and possible candidate genes see Table S4.

The ability of *Hstx2^B6^* to rescue the meiotic arrest of *Mmm*×*Mmd* F1 hybrids is subject to intrasubspecific *Mmm* polymorphisms. While asymmetric male sterility of (PWD×B6)F1 hybrids depends on the presence of the *Hstx2^PWD^* allele, another inbred strain derived from *Mmm*, known as STUS produces fully sterile F1 hybrid males with B6 mice regardless of the direction of the cross [44]. To map the STUS/PWD autosomal allelic variants that ensure full intrameiotic arrest in males carrying *Mmm* Chr X^B6^, we genotyped 84 test-cross males from crosses of B6 females with (PWD×STUS)F1 or (STUS×PWD)F1 males. QTL analysis of the sperm count (binomial, sperm cells present or absent) revealed QTLs on Chr 3, Chr 9 and Chr 13, while QTL for the testes weight mapped on Chr 3 and Chr 13 (Fig. 4). The reciprocal cross of (STUS×PWD) females with B6 males yielded only sterile male offspring without sperm in *ductus epididymis* (Table S3), strongly indicating that these autosomal QTLs interact with *Mmm* Chr X^B6^. Several interesting candidate genes with meiotic functions have been found in these QTL regions, including *Hormad1*, *Sycp1*, *H2afx* or *Msh3* (Table S4). Admittedly, 1.5-LOD support intervals of the QTLs proved quite large and none of the selected candidates displayed a dN/dS ratio indicative of their rapid evolution.

### *Hstx2* affects meiotic pairing and spermatogenic differentiation

**Figure 5:**
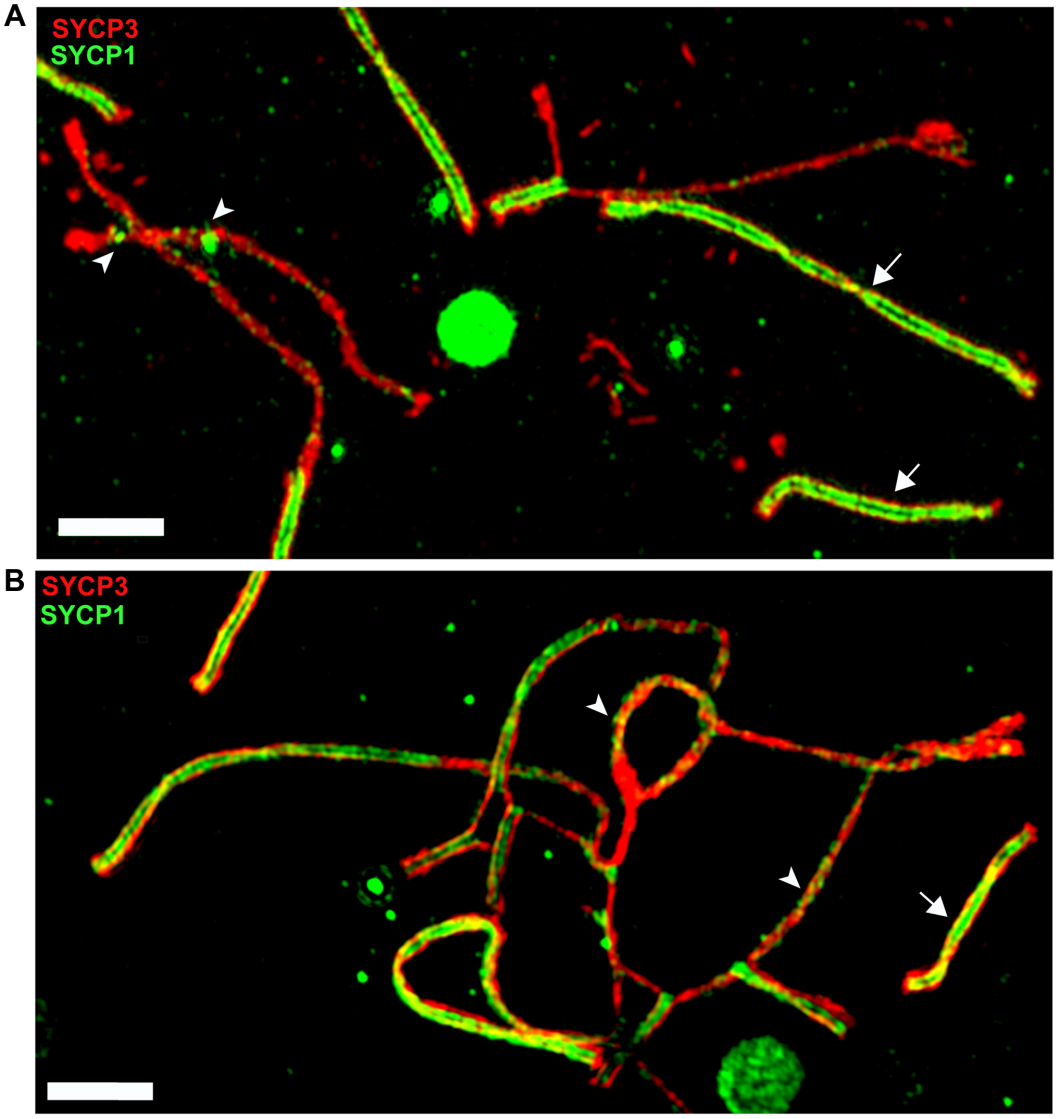
Super-resolution microscopy of synaptonemal complexes on spreads of (B6.PWD-Chr X.1s×PWD) pachytene spermatocytes. (A) Detail of a pachytene spermatocyte of a sterile male immunostained by SYCP3 (red) and SYCP1 (green) antibodies. Properly synapsed bivalents (arrows) show two parallel threads of transverse filaments decorated by SYCP1 antibody which form the central region embedded in SYCP3 lateral elements. Unsynapsed chromosomes lack transverse filaments but display some irregular SYCP1 spots (arrowheads). (B) Example of a nonhomologous pairing and/or translocations, and asynapsis in pachynema of (B6.Chr X.1s×PWD)F1 sterile male. Bar 2000 nM.

The meiotic arrest of (PWD×B6)F1 hybrid males is associated with failure of proper synapsis of homologous heterosubspecific autosomes, delay of DNA double-strand break (DSB) repair on unsynapsed autosomes and dysregulation of meiotic sex chromosome inactivation (MSCI) at the first meiotic prophase. However, full fertility and complete autosomal synapsis is restored when Chr 17 is PWD/PWD consubspecific on otherwise PWD/B6 F1 background [22]. Here we focused on the fertility parameters and pachytene chromosome synapsis in F1 hybrid males differing at the *Hstx2* locus (Fig. S4A–E). The *Hstx2^PWD^* allele in (B6.PWD-Chr X.1s×PWD) hybrid males ensured full sterility, meiotic arrest at mid-late pachynemas, almost absent diplotene spermatocytes and a lack of sperm. Immunostaining of SYCP3 and SYCP1 components of lateral and central elements of synaptonemal complexes or HORMAD2 protein revealed unsynapsed autosomes in >90% of pachynemas of both, (PWD×B6) and (B6.PWD-Chr X1s×PWD) F1 hybrid males. The super-resolution structured illumination microscopy documented irregular spots of SYCP1 on some univalents and nonhomologous synapsis and/or translocations (Fig. 5), resembling “tangles” observed in pachynemas with reduced frequency of DSBs [45]. In contrast, (B6.PWD-Chr X.1×PWD) males carrying *Hstx2^B6^* were semifertile, with partial meiotic arrest at late pachytene stage. Only 34% of pachynemas showed asynapsis of one or two pairs of autosomes (Fig. S4A–E). It can be concluded that in F1 hybrid males the *Hstx2^B6^* allele partially restores fertility and significantly reduces the frequency of pachynemas with asynapsis and the number of unsynapsed autosomes per cell.

To test whether the occurrence of meiotic asynapsis is not limited to the (PWD×B6) strain combination, we checked chromosome synapsis in pachytene spermatocytes of (STUS×B6)F1 and (PWD×SCHEST)F1 hybrids. SCHEST is a wild-derived strain of *Mmd* (J.P., unpublished). Both F1 hybrids were sterile, showing no sperm (STUS×B6) or few sperm (PWD×SCHEST, <0.9 mil.) in *ductus epididymis*. In both cases >90% of pachytene spermatocytes revealed multiple pairs of unsynapsed autosomes (Fig. S5A, B). Thus, asynapsis in *Mmm*×*Mmd* intersubspecific hybrids is a more general phenomenon, not confined to the incompatibilities between B6 and PWD genome.

### *Hstx2* and *Hst1/Prdm9* regulate male but not female asynapsis of heterosubspecific homologs

**Figure 6:**
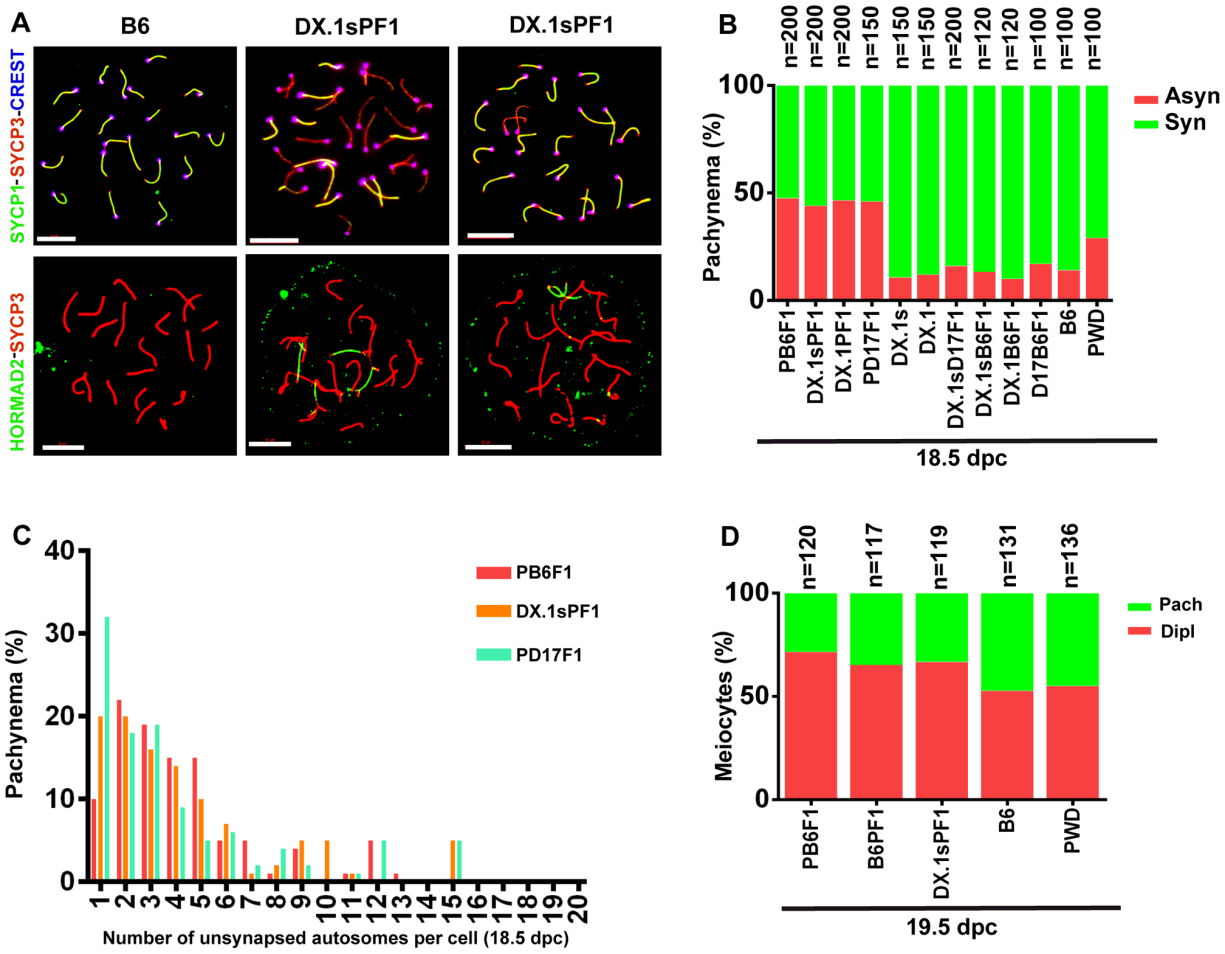
Meiotic asynapsis in female hybrids. Abbreviations of consomic strains and their hybrids: DX.1 – B6.PWD-Chr X.1; DX.1PF1 – (B6.PWD-Chr X.1×PWD)F1; DX.1sPF1 – (B6.PWD-Chr X.1s×PWD)F1; DX.1B6F1 – (B6.PWD-Chr X.1×B6)F1; DX.1sB6F1 – (B6.PWD-Chr X.1s×B6)F1; DX.1sD17F1 – (B6.PWD-Chr X.1s×B6.PWD-Chr 17)F1; D17B6F1 – (B6.PWD-Chr 17×B6)F1; B6PF1 – (B6×PWD)F1, PB6F1 – (PWD×B6)F1; PD17F1 – (PWD×B6.PWD-Chr 17)F1. (A) Chromosome synapsis in pachytene oocytes of B6 and (B6.PWD-Chr X.1s×PWD)F1 18.5–19.5 dpc female fetuses was analyzed by combination of SYCP1, SYCP3 and CREST (centromeric heterochromatin) immunostaining or by HORMAD2 and SYCP3 to detect unsynapsed chromosomes. Bar, 10 µm. (B) The frequency of oocytes showing one or more asynaptic chromosomes is similar (>40%) irrespective of *Hstx2* and *Prdm9/Hst1* genotype. (C) Although the (PWD×B6), (B6.PWD-Chr X.1s×PWD), (B6.PWD-Chr X.1×PWD) and (B6.PWD-Chr 17×PWD)F1 hybrid females do not differ in percentage of pachytene oocytes with asynapsis, the (B6.PWD-Chr 17×PWD)F1 females, conspecific for Chr 17^PWD^, carry significantly less asynapsed chromosomes per cell. (D) The frequency of diplonemas in spread oocyte preparations was significantly lower (p<0.01, χ^2^ test) in intersubspecific hybrids than in parental inbred strains..

Asynapsis preferentially affects autosomal pairs with heterosubspecific homologs, and their pairing failure is strongly influenced by the *Prdm9* and *Hstx2* genes in sterile hybrid males ([22] and above). We asked whether the genetic control of meiotic asynapsis differs between male and female gametogenesis of intersubspecific hybrids. The pachytene chromosome asynapsis was not observed in PWD and B6 spermatocytes, but occurred in 14% and 29% of pachytene oocytes of the same genotype. In (PWD×B6)F1 hybrid females, 47.5% of pachynemas showed asynapsis, but contrary to the F1 hybrid males the frequency of asynaptic oocytes was not dependent on the *Prdm9* and *Hstx2* genotypes (Fig. 6A, B). The conclusion was reached from the comparison of male and female meiosis in hybrids between particular consomics and PWD. Thus, 46% of (PWD×B6.PWD-Chr 17)F1 pachytene oocytes displayed asynapsis that was completely absent in spermatocytes of the same genotype. Moreover 46.5% and 44% of oocytes of (B6.PWD-Chr X.1×PWD)F1 and (B6.PWD-Chr X.1s×PWD)F1 hybrids showed asynaptic autosomes (Fig. 6B), compared to 34.1% and 96.4% of pachynemas of the corresponding male genotypes. It can be concluded that contrary to male meiosis, Chr 17 and *Hstx2* do not change the overall frequency of asynaptic pachynemas in female meiosis of intersubspecific hybrids. However, detailed analysis of female hybrids consubspecific for Chr 17^PWD^ showed a lower number of unsynapsed autosomes per cell (p<0.01) when compared with the other intersubspecific F1 hybrid genotypes (Fig. 6C). Thus, *Prdm9/Hst1* and/or some other genes on Chr 17 exert a limited effect on asynapsis in female hybrids as well. The elevated incidence of asynaptic oocytes predetermined to elimination in intersubspecific ovaries was reflected by a reduced ratio of diplotene/pachytene oocytes in ovarian cell spreads (Fig. 6D).

### Heterosubspecific but not consubspecific homologs are sensitized to asynapsis in female intersubspecific hybrids as well

We have shown that consubspecific (PWD/PWD) homologous autosomes evade asynapsis in otherwise heterosubspecific (PWD/B6) genomic background of hybrid males. The finding indicated a *cis*-type of asynapsis control based on some kind of mismatch between orthologous chromosomes of *Mmm* and *Mmd* origin [22]. Considering the difference between male and female hybrids in the overall frequency of asynapsis and the male-limited effect of HS genes we asked whether the mismatch of heterosubspecific homologs lowering their synapsis efficiency also operates in female meiosis.

For this purpose we analyzed primary oocytes of female hybrids between PWD and chromosome substitution strains carrying Chr 17^PWD^, Chr X.1^PWD^ or Chr X.1s^PWD^, respectively. We compared the efficacy of meiotic synapsis of consubspecific (PWD/PWD) Chr 17 and Chr X homologs with matching heterosubspecific pairs in (PWD×B6)F1 pachytene oocytes and oocytes from the parental controls. Using the whole-chromosome DNA FISH we also visualized Chrs 2, 16, 18, 19 and X. In (PWD×B6)F1 pachynemas Chr 2 showed the lowest incidence of asynapsis. The frequency of univalents of small autosomes 16, 17, 18 and 19 varied between 18% and 49% in asynaptic pachytene oocytes. Strikingly, Chr X displayed the highest frequency (64%) of asynapsis (Table 2).

**Table 2:** Asynapsis of individual chromosomes in pachytene oocytes of intersubspecific F1 hybrids and parental controls.

	Frequency of asynaptic chromosomes in pachytene oocytes^a^						
Genotype^b^	Chr 2	Chr 16	Chr 17	Chr 18	Chr 19	Chr X	Any Chr
**B6**			16.3 (2.8) n = 43			54.9 (9.3) n = 51	17.0 N = 100
**PWD**			15.9 (4.6) n = 138			55.8 (16.2) n = 138	29.0 N = 100
**DX.1sD17F1**			15.2 (2.4) n = 46			30.0 (4.8) n = 50	16.0 N = 200
**PB6F1**	9.8 (4.6) n = 102	23.0 (10.9) n = 61	17.8 (8.4) n = 146	49.4 (23.7) n = 81	30.0 (14.4) n = 60	64.0 (30.7) n = 150	48.0 N = 200
**DX.1sPF1**			25.6 (11.3) n = 156			**5.6 (2.5)** n = 178	44.0 N = 200
**PD17F1**			**0 (0)** n = 119			55.4 (25.5) n = 112	46.0 N = 150

a Each column represents the sum of two or three independent biological replicas. Asynapsis of each Chr was measured in a separate experiment in a separate set of cells. Incidence of asynapsis of a particular Chr is shown for cells with at least one asynapsis. Value in parenthesis is an estimate of overall frequency of asynapsis of a given Chr considering the overall frequency of cells with any asynapsis (Any Chr column). n - number of cells with asynapsis. N - total number of cells examined.

b Abbreviations: DX.1sD17F1 – (B6.PWD-Chr X.1s×B6.PWD-Chr 17)F1, DX.1sPF1 – (B6.PWD-ChrX 1s×PWD)F1, PD17F1 – (PWD×B6.PWD-Chr 17)F1.

The asynapsis of consubspecific Chr 17^PWD/PWD^ homologs dropped to zero in (PWD×B6.PWD-Chr 17)F1 oocytes, although the total frequency of pachynemas with asynapsis was the same as in (PWD×B6) hybrids. In (PWD×B6.PWD-Chr X.1s)F1 oocytes, 69.9 Mb of the centromeric part of Chr X was consubspecific for the PWD sequence, while the end of the chromosome, 101.4 Mb in length, was PWD/B6 heterosubspecific. Nevertheless, the partial PWD homozygosity was sufficient to reduce Chr X asynapsis from 64% down to 5.6% of pachytene oocytes (Table 2). It can be concluded that asynapsis in intersubspecific female and male hybrids follows the same rule, depending on yet unspecified sequence incompatibility between individual homologs of *Mmm* and *Mmd* origin.

### Pursuing the dominance theory of Haldane's rule

To explain Haldane's rule of hybrid sterility, the dominance theory posits the recessive nature of X-linked variants that disrupt gametogenesis in hemizygous (XY) but not in homozygous (XX) sex [9]. In its most straightforward interpretation the F1 hybrid females should be sterile in the same way as their hemizygous male sibs if their genotype were made homozygous for an incompatible Chr X variant. We constructed such genotype by crossing consomic females B6.PWD-Chr.X.1s with PWD males. The resulting female hybrids were PWD/B6 heterosubspecific for the whole autosomal genome but consubspecific for proximal 69.9 Mb of Chr X^PWD^, encompassing the *Hstx1/2* hybrid sterility locus. Contradicting the simple interpretation of Muller's dominance hypothesis the (B6.PWD-Chr.X.1s×PWD)F1 hybrid females were fully fertile, as were the parental controls (Table S5). However, the results were concordant with the testis-specific function of *Hstx2^PWD^*. Thus, to follow the dominance theory, the preponderance of male-limited HS in species with heterogametic sex could be explained by a predominance of recessive, compared to dominant, mutations of HS genes and their male-limited expression. Admittedly, the latter premise is in conflict with HS obeying the Haldane's rule in birds and *Lepidoptera*.

## Discussion

### The role of *Hstx2* in F1 hybrid sterility

Disproportionate involvement of Chr X in HS has been well documented in classical studies of *Drosophila* hybrids (for review see [46], [47]) and repeatedly reported in hybrids of the house mouse subsp [27], [33], [36], [48]. The introgression of Chr X^PWD^ of *Mmm* into *Mmd* B6 genetic background resulted in abnormal sperm morphology and inability to fertilize eggs. This phenotype is controlled mainly by the *Hstx1* locus supported by additional loci on Chr X [33]. With the aim to positionally clone the *Hstx2* gene we narrowed down the critical region to a 4.7 Mb interval (Chr X: 64.88 Mb–69.58 Mb) and showed that it also carries the *Hstx1* locus. In contrast to *Hstx1* phenotype, the intrameiotic arrest is controlled from a single *Hstx2* locus on Chr X. The *Hstx2/Hstx1* critical interval is overlapped by and may be identical with the 8.4 Mb QTL responsible for the sterilizing effect of *Mmm* Chr X^PWK^ introgressed into the genetic background of *Mmd* LEWES inbred strain [30]. Using the position on Chr X, spermatogenic expression, and dN∶dS ratio Good and coworkers [41] predicted nine candidate genes for X-linked hybrid sterility, three of which (*Ctag2*, *4933436I01Rik* and *Fmr1nb*) also occur in the present list of *Hstx1/2* candidates. Moreover, the *Sha2* locus of the Japanese house mouse *Mus m. molossinus* [49], potentially identical with *Hstx1*, maps to the same interval. *Sha2* is responsible for spermiogenic arrest in B6 males carrying Chr X*^M.m.molossinus^* introgression [49]. Among the candidate genes in the region, *Fmr1nb* and *4933436I01Rik* are expressed in the appropriate cell type during germ cell differentiation and display two and seven non-synonymous substitutions, respectively. The critical region also contains the *Mir465* cluster of miRNA genes, which show a significant difference in expression between reciprocal F1 hybrids. Moreover, *Mir743a* carries a single SNP in its seed sequence. Admittedly, the *Hstx1/Hstx2* candidate region is still too large to finalize the list of *Hstx2/Hstx1* candidate genes. Recently, many ampliconic genes on the mouse and human Chr X were shown to be unique for a given species and expressed predominantly in testicular germ cells [50]. These features make them potential candidates for reproductive isolation genes. The *Hstx1/Hstx2* critical region is flanked by amplicons *4930527E24Rik* and *Xlr* (amplicons 7 and 9 in [51]). However, none of the candidate protein-coding genes or *Mir* genes is located within a known amplicon and all protein-coding candidates have an ortholog in other mammalian species.

The incompatible alleles of major HS loci are not fixed to homozygosity within *Mmm* and *Mmd* subspecies, in agreement with the idea of the early stage of their speciation. *Hst1/Prdm9* is polymorphic for “sterility” and “fertility” alleles in natural populations and in inbred strains [23], [44], [52] and the same is true for the X-linked HS QTLs [44], [53]. The asymmetric contribution of *Mmm* and *Mmd* genomes to HS of reciprocal hybrids reveals polymorphic control as well. Chr X^B6^ causes meiotic arrest depending on PWD/STUS polymorphic modifiers on Chrs 3, 9 and 13.

The proximal part of Chr X carries loci for major incompatibilities also outside the *Mus musculus* group of mouse subpecies. Introgression of Chr X of *Mus spretus* into B6 inbred strain reduced testes weight and fertility [54], with the largest LOD score mapping approximately 10 Mb proximal to the *Hstx1/Hstx2* region. The males were semifertile, with many tubules containing normally developing spermatozoa but some tubules completely devoid of germ cells. The testes of (*Mus macedonicus*×B6) F1 hybrid males displayed premeiotic block with Sertoli cells and dividing spermatogonia. The QTL analysis of a backcross population revealed two major loci, again on Chr 17 and Chr X, but with positions distal to *Prdm9* and to *Hstx1/Hstx2* loci, respectively [55]. These crosses confirmed the large X-effect in mice but did not reveal common HS genes beyond the group of house mouse subspecies.

Laboratory crosses of wild-derived inbred strains continue to reveal new details about the genetic control and mechanistic basis of hybrid sterility, but their direct role in speciation is less clear. In an alternative approach, evolutionary biologists collect wild mice from the house mouse hybrid zone to analyze the introgression of (sub)species-specific DNA markers. Because this approach can reflect all kinds of incompatibilities restricting the gene flow, the results are usually complex, uncovering multiple regions on autosomes and Chr X. Nonetheless, at least two common themes have emerged from the field data and laboratory crosses, namely the large X-effect and a specific role of 60 Mb–80 Mb interval of Chr X in reproductive isolation of *Mmm/M. m. molossinus* and *Mmd* [16], [17], [56], [57]

### The role of meiotic chromosome asynapsis in intersubspecific reproduction barrier

More than 90% of primary spermatocytes of sterile *Mmm*×*Mmd* F1 hybrids fail to synapse properly their chromosomes at the pachytene stage of meiosis. Unsynapsed autosomes carry DMC1/RAD51 foci on unrepaired DSBs and are decorated by the phosphorylated form of histone H2AFX. The sex body containing X and Y chromosomes is often malformed or disappears, and transcriptional inactivation of sex chromosomes (MSCI) is disrupted [22], [58]. Such failure of chromosomes to synapse can be under *trans*- or *cis*-control. Mutations of various meiotic genes involved directly or indirectly in meiotic chromosome pairing and synapsis cause asynapsis of multiple autosomes *in trans* [59]. A null allele of the *Prdm9* gene on *Mmd* background causes male and female sterility associated with asynapsis and failure to form the sex body [60], the phenotype similar to sterile *Mmm*×*Mmd* hybrids [22], [24]. Null mutations of *Sycp1*, *Hormad1* or *Mei4*, the candidate genes regulating the *Hstx2*-controlled asymmetry of HS, also cause asynapsis. Contrary to genes acting *in trans*, certain structural mutations of chromosomes such as translocations or inversions can cause local, *cis*-acting asynapsis, followed by meiotic arrest and sterility [43], [61]–[63]. To distinguish between the *trans*- and *cis*- control of asynapsis in sterile *Mmm*×*Mmd* males, we modified the F1 hybrids using the intersubspecific chromosome substitution strains B6.PWD-Chr# [20]. In (B6.PWD-Chr#×PWD)F1 hybrids we compared the ability of consubspecific and heterosubspecific (PWD/PWD vs PWD/B6) chromosomes to synapse on otherwise F1 hybrid background. These experiments clearly showed that asynapsis is regulated primarily *in cis*, because heterosubspecific chromosome pairs in male as well as in female meiosis were more prone to failure to synapse. The sensitivity of heterosubspecific chromosome pairs to asynapsis was strongly modified *in trans* by the *Prdm9* and *Hstx2* HS genes because only *Prdm9^PWD/B6^* and *Hstx2^PWD^* allelic combinations on F1 hybrid background resulted in asynapsis of multiple autosomes in >90% of pachytene spermatocytes and full meiotic arrest. A few proteins required for meiotic chromosome alignment and pairing have been described [59]; however, the *cis*-acting signals which ensure pairing and synapsis of homologous chromosomes are still unknown. Our assay of meiotic synapsis, comparing the consubspecific versus heterosubspecific pairing homologs, offers a genetic approach to solving the problem. Such experiments are in progress.

### Haldane's rule and male-specific effect of *Hstx2^PWD^* in mouse hybrids

Hybrid sterility affects preferentially heterogametic sex, males in *Drosphila* or mammals, and females in birds and *Lepidoptera*. Several hypotheses have been proposed to explain Haldane's rule, including the dominance theory, the faster-male theory, the faster X theory and meiotic drive [47]. According to Muller's dominance theory, Haldane's rule can be explained by hemizygosity of the X (Z) chromosome in the heterogametic sex. Both, recessive and dominant X (Z)-linked HS genes could control HS in heterogametic sex, but only dominant HS genes could sterilize the homogametic sex. Using attached-X chromosome, Coyne and others tested the prediction by making *Drosophila* hybrid females homozygous for the recessive-acting X chromosome. In *D. simulans*×*D. mauritiana* or *D. sechellia* hybrids the female hybrids carrying two *D. simulans* Chr X were fertile, contrary to the prediction (for review see [47] and references therein). The authors argued that because HS genes are male specific, suggesting extra developmental sensitivity of spermatogenesis relative to oogenesis, their recessive forms manifest DMI in male but not in female gametogenesis. Indeed, the mouse *Hst1/Prdm9* and *Hstx2* are also male-specific HS loci, therefore not contradicting the fertility of female *Mmm*×*Mmd* F1 hybrids homozygous for the *Hstx2^Mmm^* allele. To accommodate the dominance theory with X-linked, male-specific HS genes, their recessive nature could be tested by a dominant transgene from the *Mmm* subspecies.

### A mechanistic model of F1 hybrid sterility

**Figure 7:**
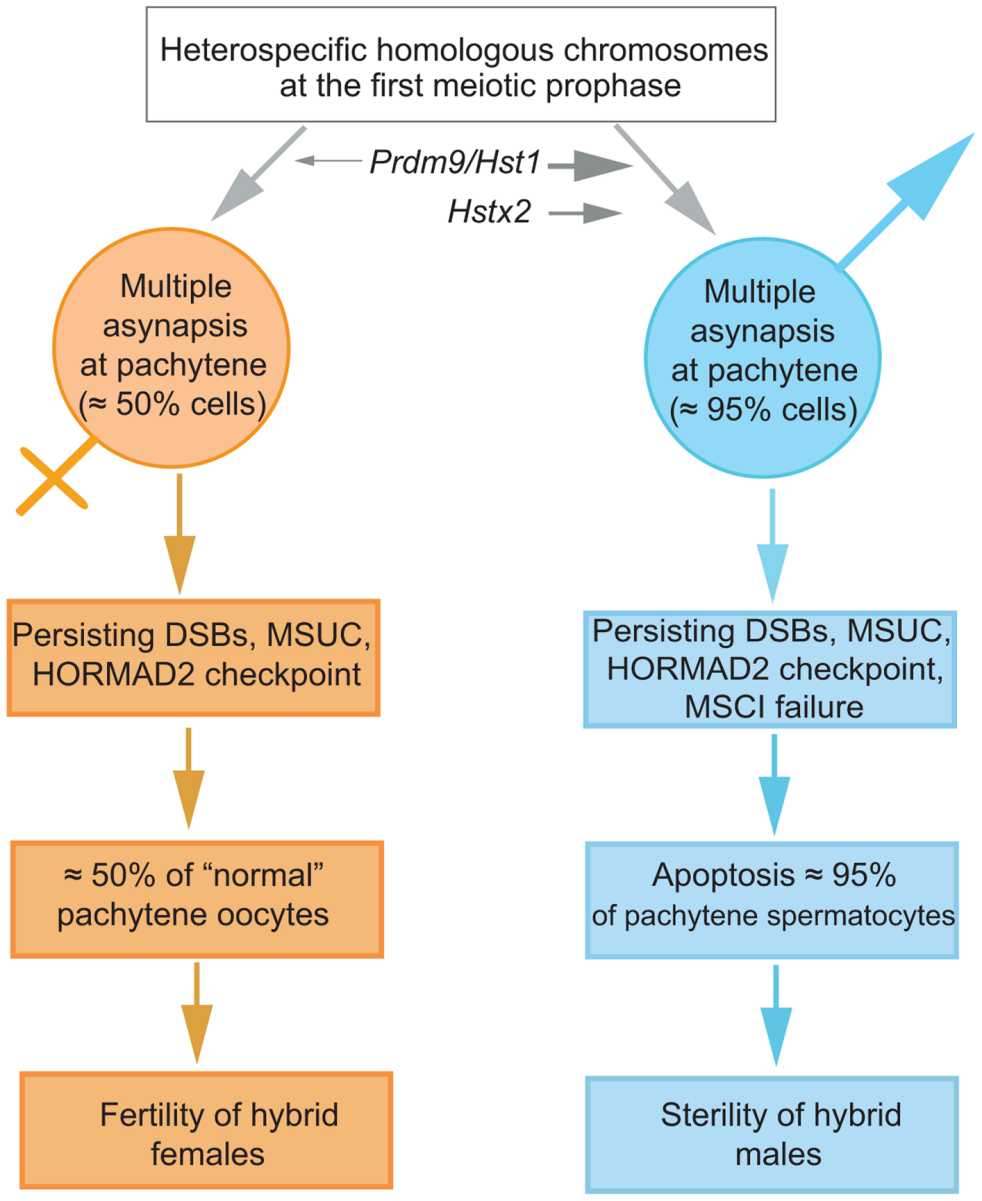
The proposed sequence of events leading to male limited sterility of intersubspecific hybrids of house mouse. Susceptibility of heterosubspecific homologs to asynapsis is common to both sexes. *Prdm9/Hst1* and *Hstx2* hybrid sterility genes can modulate this sensitivity from 0% to >95% in spermatogenesis but not in oogenesis, depending on allelic combinations of epistatic DMIs. Multiple asynaptic autosomes provoke MSCI, contributing to hybrid male sterility. Approximately one half of unaffected oocytes ensure fertility of hybrid females. It remains to be established what is the cause of asynapsis of heterosubspecific homologs.

We propose a mechanistic model of HS based on the assumption that *cis*-controlled pachytene asynapsis is the primary cause of apoptosis of primary spermatocytes and sterility in intersubspecific hybrids of the house mouse (Fig. 7). We have found that the number of unsynapsed autosomes per cell varies, indicating that the same type of *cis*-acting mechanism operates on individual autosomes. Although the molecular mechanism of homologous chromosome recognition during meiotic pairing and synapsis is unknown in mammals, one possibility is that fast evolving noncoding DNA and/or RNA sequences interfere with homology search of single-strand 3′ends on heterosubspecific homologs during DSB repair, thus interfering with their synapsis during the first meiotic prophase (see also [45]). Alternatively, the process of homolog recognition can be affected earlier, before meiotic recombination begins.

The male-specific action of the *Hstx2* or *Prdm9/Hst1* HS genes could explain why the same perturbation of sequence homology acts differently in male and female meiosis. While in female pachynemas the percentage of meiocytes with asynaptic chromosomes ranges between 40% and 45% and is ultimately unaffected by the *Hstx2* or *Prdm9/Hst1* genes, meiotic asynapsis of F1 males depends considerably on their genotype. Full sterility and complete meiotic block is associated with the *Prdm9^PWD/B6^ Hstx2^PWD^* genotype only, while the substitution of *Hstx2^PWD^* for *Hstx2^B6^* on otherwise F1 hybrid background reduces the incidence of cells with asynapsis to the level found in female meiosis. Moreover, full recovery of fertility and meiotic pairing can be achieved in F1 hybrid males consubspecific for Chr 17^PWD^ (homozygous *Prdm9^PWD/PWD^*). We expect that analysis of recombinant heterosubspecific/consubspecific chromosome pairs could provide the genetic approach to identifying the *cis*-acting sites important for meiotic pairing and synapsis.

## Materials and Methods

### Animals and ethics statement

The animals were maintained at the Institute of Molecular Genetics in Prague and Institute of Vertebrate Biology in Studenec (Academy of Sciences), Czech Republic. The project was approved by the Institutional Animal Care and Use Committee of the Institute of Molecular Genetics, AS CR, protocol No. 141/2012. The principles of laboratory animal care (NIH Publication No. 85-23, revised 1985) as well as specific Czech Law No. 246/1992 Sb. compatible with the corresponding EU regulations and standards, namely Council Directive 86/609/EEC and Appendix A of the Council of Europe Convention ETS123, were observed.

C57BL/6J (B6, mostly *M. m. domesticus*) originated from The Jackson Laboratory (Bar Harbor, ME). The PWD/Ph (PWD) and STUS strains were derived from wild *M. m. musculus* [32], [53]. The chromosome substitution (consomic) strains C57BL/6J-Chr #^PWD^ [20], abbreviated here B6.PWD-Chr #, were maintained in a pathogen-free barrier facility with a 12 h light/12 h dark cycle. The mice had *ad libitum* access to a standard rodent diet (VELAZ, ST-1, 3.4% fat) and acidified water. All males were sacrificed at the age of 60 to 70 days.

### Genotyping, phenotyping and histology

All 124 male mice from (B6.PWD-Chr X×B6)F1×PWD cross and 71 male progeny from (B6.PWD-Chr X×B6)F1x B6 cross were genotyped using SSLP Chr X markers listed in Table S6. Genomic DNA from mouse tails and spleens was prepared by the HotSHOT method [64] followed by phenol-chloroform cleaning [65]. For genotyping B6.PWD-Chr X# partial consomics and 84 males of B6×(PWD×STUS) test-cross we used MegaMUGA high-density genotyping array carrying 77,000 markers on Illumina Infinium Platform (http://csbio.unc.edu/CCstatus/index.py).

Eight weeks old male progeny of the crosses were phenotyped for testes weight (TW), sperm count (SC) and percentage of abnormal spermatozoa as described [33]. The B6×(PWD×STUS) males were phenotyped at 60 days of age as described [44]. For histological analysis the paraffin-embedded testicular sections were stained with periodic acid Schiff and hematoxylin-eosin and observed using a Nikon Eclipse 200 microscope. The Penguin 150CL CCD color camera (Pixera) was used to capture photographs, which were processed using Adobe Photoshop (Adobe Systems).

### QTL analysis

QTL mapping of recombinant males from (B6.PWD-Chr X×B6)F1×PWD and B6×(PWD×STUS) crosses was performed using the R 12.1 and its R/qtl package [66] Marker positions were taken from MGI mouse genetic map [67]. Standard interval mapping was implemented using *scanone* function. TW and logSC were modeled as continuous variables, fertility/sterility as a binary variable. Genotype probabilities between markers were calculated at a grid size of 5 cM and with genotyping error rate of 0.01%. Genome-wide significance was calculated by 1000 permutations and compared to α = 5% threshold.

### Fluorescence-activated cell sorting of spermatogenic populations

Three spermatogenic populations (leptotene+zygotene+early pachytene, mid-late pachytene+diplotene, and spermatids) were isolated using fluorescence-activated cell sorting (FACS) as described earlier [43], [68] from PWD and B6 testes. The cells were directly sorted into QIAzol lysis reagent of the miRNeasy Mini isolation kit (QIAGEN). Small aliquots of cells were sorted in Krebs-Ringer bicarbonate medium for indirect immunofluorescence analysis. The population composition was estimated by staining with anti-SYCP3, anti-SYCP1, anti-γH2AFX antibodies (details below) and DAPI (Vectashield). All sorted populations showed 85–90% purity of the desired cell type.

### Microarray miRNA and protein-coding gene transcription analysis and qRT-PCR validation

Total RNA was isolated from sorted cells and 14.5 dpc testis using the miRNeasy Mini isolation kit (QIAGEN) as recommended. RNA concentration was determined by NanoDrop (NanoDrop Technologies) and its integrity checked in Agilent 2100 bioanalyzer, RNA Lab-On-a-Chip (Agilent Technologies). The total RNA (20–30 ng for gene expression and 120 ng for miRNA expression) was converted to cRNA using the Affymetrix Two-Cycle Target Labeling kit according to the manufacturer's instructions or using the Affymetrix 3′ IVT Express Kit. Affymetrix GeneChip Mouse Genome 430.2.0 array, and Affymetrix GeneChip miRNA 1.0 Array was hybridized with cRNA. The data obtained from the experiments were analyzed using Bioconductor [69] (http://www.bioconductor.org/) and the R project for statistical computing (version 2.12; http://www.r-project.org/). The probes were annotated to Ensembl gene identifiers using the custom chip description file, which was based on NCBI build 37. The data were normalized using RMA (Affymetrix GeneChip Mouse Gene 1.0ST Array) and quantile normalization (Affymetrix GeneChip miRNA 1.0). We used Linear Models for Microarray Data Package, limma version 3.6 [70] for statistical evaluations of expression differences as described [22]. The microarray dataset is deposited in the NCBI Gene Expression Omnibus (GEO) with series accession number GSE41707 [22] GSE49442 and GSE49443. Expression of different X-linked protein-coding genes on spermatogenic populations were derived from NCBI GEO profiles or NCBI GEO database GSE7306 [43] and GSE49444.

For qRT-PCR of protein-coding genes, reverse transcription of isolated RNA samples was carried out using Applied Biosystems (ABI) high-capacity cDNA reverse transcription kit. The quantification of mRNAs was performed using FastStart DNA Master SYBR Green I kit (Roche) and amplified in LightCycler 2000 (Roche). Reactions without reverse transcriptase were utilized as negative control. The assays were done in biological and technical triplicates. The data were analyzed using LightCycler Software version 3.5.3 (Roche). For validation of miRNA expression we used ABI TaqMan MicroRNA assays and followed the manufacturer's instructions. The reactions were cycled in Applied Biosystems 7300 Real-time PCR system, and associated software was used for data analysis. The reactions were also carried out using biological and technical triplicates and proper negative controls. The highest and stably expressed miRNAs, U6 non-coding RNA for sorted cells and Mir152 for 14.5 dpc testis, were used as the reference for data normalization. The primers were designed using Primer 3 software (http://frodo.wi.mit.edu/). Sequences of primers are given in Table S6.

### Sequencing, SNP and PWD exome analysis

Exome sequence analysis was carried out for PWD/Ph mice at BGI Europe using Illumina HiSeq 2000 sequencers. The PWD exome sequence was aligned to NCBIm37 genome (BAM format, http://samtools.sourceforge.net/SAM1.pdf) and deposited at the Sequence Read Archive (SRA) accession SRR942524.

All the non-synonymous mutations between PWD and B6 for 4.7 Mb *Hstx2* locus were tabulated. Sequence validations on PWD cDNA (for protein-coding genes) and PWD BACs (for miRNAs) [35] were carried out as described [24] using sequencing capillary machine ABI310 (Applied Biosystems). The sequences of primers are listed in Table S3. Some of the SNPs were also confirmed using the Mouse Phenome database (http://phenome.jax.org/). The dN∶dS ratio (an indicator of evolutionary selective pressure on genetic processes) of different X-linked protein-coding genes between rat and mouse was calculated using Ensemble Biomart.

### Immunostaining and DNA FISH of spread meiocytes

The meiocyte spreads were prepared by using the hypotonic protocol as described earlier [21], [71]. The nuclei were immunostained using following primary antibodies; rat polyclonal anti-SYCP3 (Abcam, #15092), mouse monoclonal anti-SYCP1 (Abcam, #15087), guinea pig anti-histone linker H1t [72], human autoimmune anti-centromere (AB-Incorporated, #15-235), mouse monoclonal anti-γH2AFX (Upstate, #05-636), mouse monoclonal antibody anti-SYCP3(D-1) (Santa Cruz #74569), rabbit polyclonal antibody HORMAD2(C-18) (Santa Cruz #82192) and the secondary antibodies: goat anti-Rabbit IgG-AlexaFluor488 (Molecular Probes, A -11034), goat anti-Mouse IgG-Alexa Fluor 568 (Molecular Probes, A-11031), goat anti-Rabbit IgG-Alexa Fluor 568 (Molecular Probes, A-11036), goat anti-Mouse IgG-Alexa Fluor 350 (Molecular Probes, A- 21049), goat anti-Mouse IgG-Alexa Fluor 647 (Molecular Probes, A-21236), goat anti-Rabbit IgG-Alexa Fluor 647 (Molecular Probes, A-21245) and goat anti-Guinea pig IgG-Cy3 (Chemicon, #AP108C). The immunolabeled meiocytes were subjected to DNA FISH using Metasystems XMP XCyting Mouse Chromosome N Whole Painting Probes for Chrs 2, 16, 17, 18, 19 and X as described [73]. The images were acquired using a Nikon Eclipse 400 (Tokyo, Japan) microscope with motorized stage control using a Plan Fluor objective, 60× (Nikon, MRH00601) and captured using a DS-QiMc monochrome CCD camera (Nikon) and NIS elements program. The fluorescent intensity of images was adjusted using Adobe Photoshop CS software (Adobe Systems). For super-resolution microscopy the meiotic spreads were examined with the Zeiss Axioimager Z.1 platform equipped with the Elyra PS.1 super-resolution system for SR SIM and the LSM780 module for CLSM, using Alpha Pln Apo 63×/1.40 oil Zeiss objective (total magnification 1008×) with appropriate oil (Immersol 518F, by Zeiss). SR-SIM setup was 5 rotations and 5 phases for each image layer. Up to 7 (usually 3) 110 nm Z-stacks were acquired per image. Staging of meiotic prophase I in males and females were done as described earlier [22].

### Statistics

Multiple biological replicates of each genotype were analyzed for cellular phenotypes and RNA expression. The significance of body weight, testes weight, sperm morphology and breeding phenotypes was computed using Welsch's t-test. Sizes of chromatin areas covered by the hybridization signal in synapsed and unsynapsed autosomes were compared by Analysis of Variance (ANOVA) with Tukey's correction for multiple testing. Differences between cellular phenotypes were determined with χ2 test. All computations were done using R 2.15.0 or Graphpad Prism (http://www.graphpad.com/scientific-software/prism/#1).

## Supporting Information

Figure S1 Mapping of *Hstx1* locus on Chr X. The fertility of males with PWD/B6 single recombination declined with the recombination breakpoints in the interval *DXMit76* (64.75 Mb) and *DXMit143* (69.28 Mb), indicating the position of the *Hstx1* gene.
(EPS)

Figure S2 Gene map of the *Hstx2/Hstx1* critical region on Chr X. Predicted genes and pseudogenes are not included in this map. See text for details.
(EPS)

Figure S3 Expression profiling of protein-coding genes within *Hstx1/Hstx2* critical region. (A) Log fold-change of expression ratio of PWD versus B6 protein-coding genes in flow sorted testicular cells. Data from Affymetrix GeneChip Mouse Gene 1.0ST Array do not show any significant differences between both strains in expression levels in primary spermatocytes. Abbreviations: Z-EP – Zygotene Early Pachytene, M-LP - Mid-Late Pachytene, ST – Spermatids. (B) qRT PCR of expression levels of six candidate genes in testes of 14.5 d reciprocal F1 hybrid males (PWD×B6)F1 and (B6×PWD(F1). None of the differences is significant.
(EPS)

Figure S4 The effect of *Hstx2* on spermatogenic differentiation and synapsis of meiotic chromosomes. (A) Histological crossections of spermatogenic tubules of B6 control and (B6.PWD-Chr X.1×PWD)F1, abbreviated here DX.1PF1, and (B6.PWD-Chr X.1s×PWD)F1, abbreviated DX.1sPF1 hybrids. Note the vacuolar degeneration, heteropycnotic cells and absence of sperm cells in DX.1sPF1 hybrids. (B) Unsynapsed autosomes and sex chromosomes visualized by HORMAD2 and SYCP3 immunostaining. Early pachynemas with high and low frequency of autosomal univalents are shown. (C) Frequency of primary spermatocytes at various stages of meiotic progress in males of different genotype. (PWD×B6)F1, abbreviated PB6F1, and B6.PWD-Chr X.1s×PWD)F1 (abbreviated DX.1sPF1) sterile males display the same profile of primary spermatocyte stages. (D, E) Frequency of pachynemas with asynapsis and number of unsynapsed autosomes per cell are similar in PB6F1 and DX.1sPF1 sterile males. In DX.1PF1 males asynapsis occurs with lower frequency and the pachynemas with asynapsis have lower number of univalents.
(EPS)

Figure S5 Pachytene asynapsis in sterile F1 hybrid males prepared by different combinations of *Mmm* and *Mmd* inbred strains. (A) Over 90% of pachytene spermatocytes in all three strain combinations ((PWD×B6)F1 – abbreviated PB6F1, (STUS×B6)F1 – STB6F1 and (PWD×SCHEST)F1 - PSCHF1) carry one or more asynapsed chromosome pair. Frequency of asynapsis of four analyzed autosomes varied between genotypes. Average number of univalents per cell was highest in PSCHF1 and lowest in PB6F1. (B) Distribution of pachynemas with a given number of unsynapsed chromosome pairs. Asynapsis varies widely within each genotype. A significant excess of cells with one unsynapsed pair occurs in B6PF1 hybrids.
(EPS)

Table S1 Coordinates of the introgressed PWD sequence in B6.PWD-Chr X subconsomics (GRCmm38).
(DOCX)

Table S2 Meiotic stages of adult testes of B6.PWD-Chr.X# and PWD hybrids.
(DOCX)

Table S3 Testes weight and sperm count in male progeny of B6×(STUS×PWD), B6×(PWD×STUS) and reciprocal crosses (STUS×PWD)×B6, and (PWD×STUS)×B6.
(XLSX)

Table S4 Intervals and candidate genes of Chr X^PWD^-independent HS QTLs.
(DOCX)

Table S5 Fertility of parental and F1 hybrid females with different allelic combinations at *Hstx1/Hstx2* loci.
(DOCX)

Table S6 Primers.
(XLS)
