# Supplementary material for: X Chromosome Control of Meiotic Chromosome Synapsis in Mouse Inter-Subspecific Hybrids
Source: PLoS Genet. 2014 Feb 6;10(2):e1004088. doi: 10.1371/journal.pgen.1004088 (PMC3916230; doi:10.1371/journal.pgen.1004088)
Supplement: Table S1 — Coordinates of the introgressed PWD sequence in B6.PWD-Chr X subconsomics (GRCmm38). (DOCX) [file pgen.1004088.s006.docx]

**Table S1. Coordinates of the introgressed PWD sequence in B6.PWD-Chr X subconsomics (GRCmm38).**

| **Subconsomic** | **Proximal Marker** | **Position (bp)** | **Distal Marker** | **Position (bp)** | **PWD segment (bp)** |
| --- | --- | --- | --- | --- | --- |
| B6.PWD-Chr X.1 | JAX00708539 | X:5,454,831 | UNC30904273 | X:64,880,641 | 59,425,810 |
| B6.PWD-Chr X.1s | JAX00708539 | X:5,454,831 | UNC30934795 | X:69,584,093 | 64,129,262 |
| X.1-X1s interval |  |  |  |  | 4,703,453 |
| B6.PWD-Chr X.2 | JAX00178139 | X:38,534,283 | UNC200687147 | X:129,453,937 | 90,919,654 |
| B6.PWD-Chr X.3 | DXMit114 | 98,146,308 | DXMit31 | X:163,975,834 | 65,829,526 |
