# Supplementary material for: X Chromosome Control of Meiotic Chromosome Synapsis in Mouse Inter-Subspecific Hybrids
Source: PLoS Genet. 2014 Feb 6;10(2):e1004088. doi: 10.1371/journal.pgen.1004088 (PMC3916230; doi:10.1371/journal.pgen.1004088)
Supplement: Table S2 — Meiotic stages of adult testes of B6.PWD-Chr.X# and PWD hybrids. (DOCX) [file pgen.1004088.s007.docx]

**Table S2. Meiotic stages of adult testes of B6.PWD-Chr.X# and PWD hybrids.**

| **Strain/hybrid** | **Age (weeks)** | **n** | **LEP %** | **ZYG %** | **EP %** | **MP %** | **LPD %** |
| --- | --- | --- | --- | --- | --- | --- | --- |
| B6 | 8 | 159 | 5.7 | 8.2 | 16.4 | 40.9 | 28.9 |
| (PWD x B6)F1 | 8 | 200 | 18.0 | 21.0 | 43.0 | 16.0 | 2.0 |
| (B6.PWD -Chr.X.1 x PWD)F1 | 8 | 125 | 3.2 | 7.2 | 36.0 | 28.0 | 25.6 |
| (B6.PWD -Chr.X.1s x PWD)F1 | 8 | 138 | 15.9 | 27.5 | 39.1 | 15.9 | 1.4 |

LEP, ZYG – leptonema, zygonema; EP, MP – early, mid- pachynema; LPD – late pachynema, diplonema
