# Supplementary material for: X Chromosome Control of Meiotic Chromosome Synapsis in Mouse Inter-Subspecific Hybrids
Source: PLoS Genet. 2014 Feb 6;10(2):e1004088. doi: 10.1371/journal.pgen.1004088 (PMC3916230; doi:10.1371/journal.pgen.1004088)
Supplement: Table S4 — Intervals and candidate genes of Chr XPWD-independent HS QTLs. (DOCX) [file pgen.1004088.s009.docx]

**Table S4.** **Intervals and candidate genes of Chr X^PWD^-independent HS QTLs.**

| **Chr** | **Interval (Mb, GRCm38)** | **Candidate genes** |
| --- | --- | --- |
| 3 | 65.0-135.3 | *Ccna1, Smc4, Mnd1, Hormad1, Sycp1* |
| 9 | 40.5-98.8 | *H2afx,Rbm7, 2410076I21Rik, Mns1, Mei4, Xrn1* |
| 13 | 11.4-115.9 | *Spin1, Cks2, Trip13, Msh3, Ddx4* |
