# Supplementary material for: X Chromosome Control of Meiotic Chromosome Synapsis in Mouse Inter-Subspecific Hybrids
Source: PLoS Genet. 2014 Feb 6;10(2):e1004088. doi: 10.1371/journal.pgen.1004088 (PMC3916230; doi:10.1371/journal.pgen.1004088)
Supplement: Table S5 — Fertility of parental and F1 hybrid females with different allelic combinations at Hstx1/Hstx2 loci. (DOCX) [file pgen.1004088.s010.docx]

**Table S5. Fertility of parental and F1 hybrid females with different allelic combinations at *Hstx1/Hstx2* loci.**

| **Strain or cross** | **Age in weeks** | **No. of mated females** | ***Hstx1/Hstx2*** | **Average litter size** | **No. of litters** |
| --- | --- | --- | --- | --- | --- |
| C57BL/6J (B6) | 8 | 8 | B6/B6 | 8.2 ± 1.3 | 12 |
| B6.PWD-Chr X.1 | 8 | 6 | B6/B6 | 8.5 ± 1.3 | 12 |
| B6.PWD-Chr X.1s | 8 | 6 | PWD/PWD | 8.2 ± 1.4 | 12 |
| (PWD x B6)F1 | 8 | 6 | PWD/B6 | 7.7 ± 1.0 | 12 |
| (B6 x PWD)F1 | 8 | 6 | B6/PWD | 8.0 ± 1.3 | 12 |
| (B6.PWD-ChrX.1 x PWD)F1 | 8 | 6 | B6/PWD | 7.6 ± 1.0 | 12 |
| (B6.PWD-ChrX.1s x PWD)F1 | 8 | 6 | PWD/PWD | 8.9 ± 1.6 | 12 |
| (PWD x B6)F1 | 24 | 4 | PWD/B6 | 7.2 ± 1.3 | 6 |
| (B6.PWD-Chr X.1s x PWD)F1 | 24 | 4 | PWD/PWD | 8.3 ± 1.9 | 6 |
